# Selectivity through discriminatory induced fit enables switching of NAD(P)H coenzyme specificity in Old Yellow Enzyme ene‐reductases

**DOI:** 10.1111/febs.14862

**Published:** 2019-05-13

**Authors:** Andreea I. Iorgu, Tobias M. Hedison, Sam Hay, Nigel S. Scrutton

**Affiliations:** ^1^ Manchester Institute of Biotechnology and School of Chemistry Faculty of Science and Engineering The University of Manchester UK

**Keywords:** coenzyme specificity, ene‐reductases, enzyme kinetics, Old Yellow Enzymes, rational enzyme design

## Abstract

Most ene‐reductases belong to the Old Yellow Enzyme (OYE) family of flavin‐dependent oxidoreductases. OYEs use nicotinamide coenzymes as hydride donors to catalyze the reduction of alkenes that contain an electron‐withdrawing group. There have been many investigations of the structures and catalytic mechanisms of OYEs. However, the origin of coenzyme specificity in the OYE family is unknown. Structural NMR and X‐ray crystallographic data were used to rationally design variants of two OYEs, pentaerythritol tetranitrate reductase (PETNR) and morphinone reductase (MR), to discover the basis of coenzyme selectivity. PETNR has dual‐specificity and reacts with NADH and NADPH; MR accepts only NADH as hydride donor. Variants of a β‐hairpin motif in an active site loop of both these enzymes were studied using stopped‐flow spectroscopy. Specific attention was placed on the potential role of arginine residues within the β‐hairpin motif. Mutagenesis demonstrated that Arg130 governs the preference of PETNR for NADPH, and that Arg142 interacts with the coenzyme pyrophosphate group. These observations were used to switch coenzyme specificity in MR by replacing either Glu134 or Leu146 with arginine residues. These variants had increased (~15‐fold) affinity for NADH. Mutagenesis enabled MR to accept NADPH as a hydride donor, with E134R MR showing a significant (55‐fold) increase in efficiency in the reductive half‐reaction, when compared to the essentially unreactive wild‐type enzyme. Insight into the question of coenzyme selectivity in OYEs has therefore been addressed through rational redesign. This should enable coenzyme selectivity to be improved and switched in other OYEs.

**Abbreviations**
FMN: flavin mononucleotide
MR: morphinone reductase
OYE: Old Yellow Enzyme
PETNR: pentaerythritol tetranitrate reductase
RHR: reductive half‐reaction

## Introduction

In recent years, there has been a gradual shift away from traditional synthetic methods to more environmentally friendly and sustainable approaches in the production of fine chemicals 1, 2, 3, 4. The development of novel chemoenzymatic approaches for the manufacturing of high value chemicals is driven by the ever‐increasing knowledge of enzyme structures and mechanisms, coupled with advances in metabolic engineering and synthetic biology. The asymmetric reduction of activated C=C bonds is one of the most widely employed chemical reactions in industry for which biocatalytic routes are intensively explored 5, 6. The stereoselective reduction of alkenes that contain an electron‐withdrawing group is catalyzed by a large family of enzymes, known collectively as ‘ene‐reductases’ 7, 8, 9. The majority of ene‐reductases are homologs of the Old Yellow Enzyme (OYE) family of oxidoreductases 9, 10, a large family of flavin mononucleotide (FMN)‐dependent enzymes that use NADH and/or NADPH coenzymes as ancillary hydride donors 10, 11, 12, 13. Extensive research has contributed to a wide range of catalysis applications employing OYEs, including their use in individual biocatalytic reactions 14, as components of multiple enzymatic 15, 16, 17, 18, 19 and chemoenzymatic cascade reactions 20, and in whole‐cell biotransformation reactions 21. These studies have also driven the development of effective nicotinamide coenzyme recycling systems 22, 23, 24, biomimetic counterparts, and the use of coenzyme‐independent reduction methods 25, 26, 27, 28, 29. In particular, the use of coenzyme biomimetics and coenzyme‐free reduction systems has attracted recent attention for biocatalytic reductions. However, their utilization is limited in cell factory engineering applications, where natural coenzymes are required to drive flux through natural and engineered metabolic pathways, enable coenzyme cycling and maintain redox balance. In these cases, there is a need to use self‐sufficient closed‐loop recycling systems and to have the ability to engineer predictably coenzyme specificity to meet pathway and cellular requirements.

**Figure 1:**
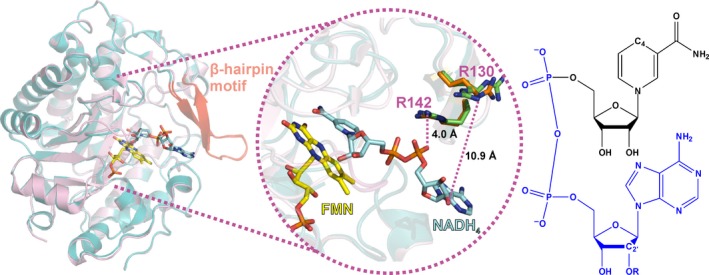
Overlaid structures of coenzyme‐free and coenzyme‐bound pentaerythritol tetranitrate reductase. The structures of oxidized PETNR (PDB: 5LGX) and PETNR:NADH_4_ complex (PDB: 3KFT) are shown as pink and teal cartoons, respectively, with the β‐hairpin structural motif highlighted in red. The middle panel encompasses a more detailed view of the active site and the β‐hairpin motif, with the FMN cofactor (yellow), the NADH_4_ coenzyme mimic (blue) and two arginine residues from the β‐hairpin motif (green in holoenzyme, orange in coenzyme‐bound PETNR) highlighted as sticks. NAD(P)H structure is shown in the right panel [R=H (NADH) or R=PO_3_^2‐^ (NADPH)], with the ‘tail’ moiety shown in blue and key atoms labeled.

Broadening of substrate scope for asymmetric bioreductions and improvements in the chemo‐, regio‐, and stereoselectivity of target compounds has been extensively reported with OYEs 15, 30, 31, 32, 33, 34, 35, 36, 37, 38, 39. However, despite these achievements, there is little understanding of the basis of coenzyme binding and selectivity in OYEs. Most use NADPH as the preferred hydride donor, but several display higher affinity and/or reactivity with NADH 40, 41, 42. Conversely, others can use both nicotinamide coenzymes (NADH and NADPH) 26. The sequence identity across different members of the OYE family is not generally conserved (< 15% conserved residues across all three classes of OYEs), and quaternary structures range from monomers to dodecamers 10. However, most OYE enzymes share a highly conserved monomer architecture, the (α,β)_8_‐barrel structure (also known as a TIM fold 43, 44), with the FMN cofactor bound noncovalently at the C‐terminal region of the β‐strands (Fig. 1). Despite this similarity, amino acid residues and/or structural motifs that direct coenzyme specificity are not known. In other dehydrogenases/reductases (e.g. based on the Rossmann fold), coenzyme discrimination is driven in part by interactions with the adenine 2′‐phosphate (NADPH) or the adenine 2′‐hydroxyl (NADH; see Fig. 1 for numbering) 45, 46. Recent studies have also suggested that coenzyme specificity can be engineered through heuristic‐based approaches involving structure‐guided, semirational strategies for enzyme engineering 47. In the ene‐reductase class, X‐ray crystallographic structures are available for several OYEs in complex with reduced coenzyme mimics [e.g., 1,4,5,6‐tetrahydro‐NAD(P), (NAD(P)H_4_)], but insight from these structures is limited. While the stacked arrangement of the nicotinamide moiety of NAD(P)H and the FMN isoalloxazine ring is conserved across these structures, the coenzyme ‘tail’ (Fig. 1) is often disordered, or in different conformations, some artificially induced by coenzyme–coenzyme stacking interactions *in crystallo*. It is this ‘tail’ that differs between NADH and NADPH, and its interaction(s) with the enzyme underpins the molecular basis of coenzyme selectivity.

Recently, we have reported the first and only structural NMR assignments of an OYE family member, pentaerythritol tetranitrate reductase (PETNR) 48, 49. PETNR is a widely studied ene‐reductase with a broad substrate scope. It is a monomeric 40 kDa enzyme, which uses both NADH and NADPH, but reacts preferentially with NADPH 50, 51, 52. Like all OYEs, the reaction catalyzed by PETNR occurs by a single‐site ping‐pong mechanism comprising a reductive half‐reaction (RHR; hydride transfer from the C4 *pro*‐R hydrogen atom of NAD(P)H to the FMN N5 atom) and an oxidative half‐reaction (hydride transfer from the FMN N5 and proton transfer from solvent to an oxidizing substrate, typically an‐α,β unsaturated alkene) 53, 54. A range of NMR chemical shift perturbations in the enzyme active site were observed upon coenzyme binding. However, these data also revealed a large reorientation of a β‐hairpin structural motif (residues T129–T147; Fig. 1) upon coenzyme binding, indicative of an induced fit mechanism. Major chemical shift perturbations were observed in particular for T131 and the neighboring R130, with more pronounced effects with NADPH_4_ compared to NADH_4_ 49. This coenzyme‐specific binding by induced fit contrasts with a previous X‐ray crystal structure, which now appears to be in an ‘open’ conformation 50.

Informed by the NMR studies, we set out to determine the molecular basis of coenzyme recognition in PETNR. We then attempted to rationally tune/switch coenzyme specificity by protein engineering of the dual‐specificity PETNR and the related NADH‐dependent morphinone reductase (MR) 42, 55. In doing so, we have identified the structural determinants of coenzyme specificity in these OYEs in a flexible and poorly conserved coenzyme‐binding pocket. On the basis of the rational engineering reported, we also suggest how protein engineering could be used to tune coenzyme specificity across other OYE oxidoreductases to facilitate future applications in biocatalysis and cell factory engineering.

## Results and Discussion

### Residue Arg130 in the β‐hairpin flap governs PETNR specificity toward NADPH

**Figure 2:**
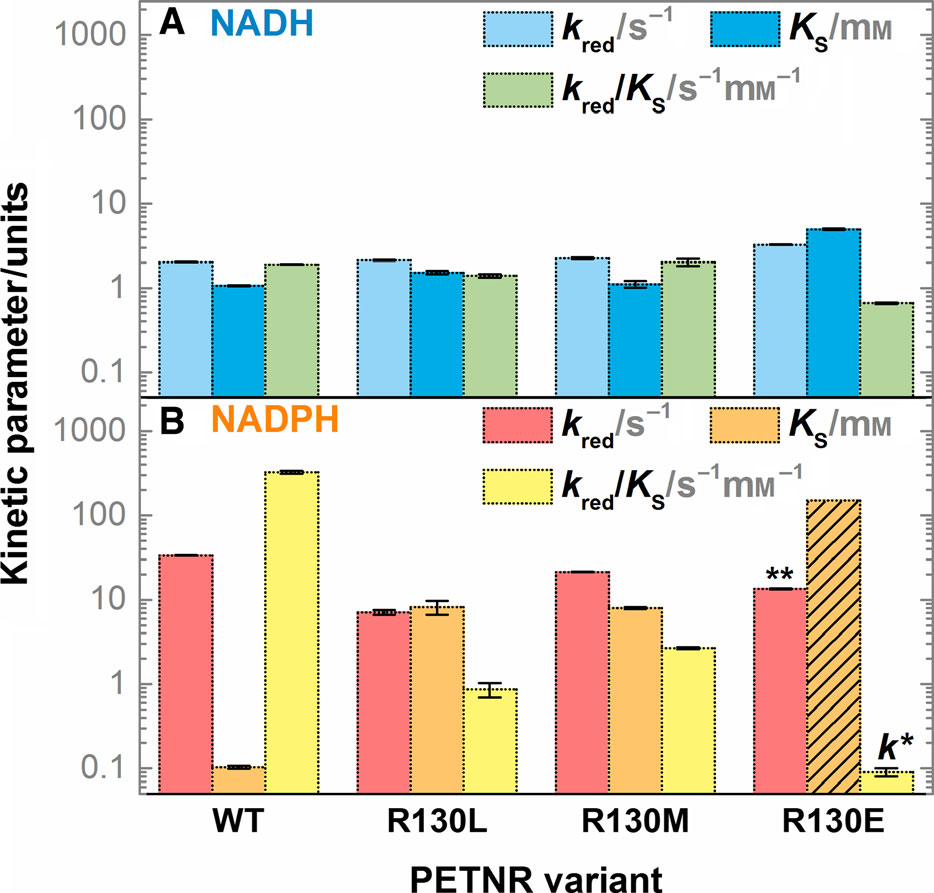
Kinetic parameters for the RHR of WT, R130L, R130M, and R130E PETNR variants with (A) NADH and (B) NADPH. The kinetic parameters are represented as bars, with the same logarithmic *y*‐axis maintained in both panels for a better comparison. *The kinetics of FMN reduction in R130E PETNR with NADPH follow a second‐order reaction, with a rate constant, *k*, represented instead of the *k*_red_/*K*_S_ value. **In this case, the *k*_red_ constant has an approximate upper limit value calculated by multiplying the second‐order rate constant (*k*) by the maximum solubility limit of NADPH in solution (*K*_S_ ~ 150 mm), and the *K*_S_ value representing the solubility limit is shown as bars crossed out with black lines. Error bars are standard errors from the fit. All kinetic parameters are tabulated in Tables S1 and S2.

Recent ^1^H‐^15^N TROSY NMR studies of the PETNR:NAD(P)H_4_ complexes have suggested a potential role for residue Arg130 in differentially binding NADPH and NADH. Aside from the perturbations observed in localized areas of the active site upon binding of either NADH_4_ or NADPH_4_, significant chemical shift perturbations are also observed in the β‐hairpin structural motif (Fig. 1) 49. Within this β‐hairpin flap, noteworthy differences in chemical shift were observed between the two complexes, indicating each coenzyme alters the orientation of the structural motif in different ways, possibly suggesting an interaction between Arg130 and the 2′‐phosphate of NADPH. However, upon inspection of X‐ray crystal structures of PETNR and NADH_4_‐bound PETNR (Fig. 1), an interaction between Arg130 and the bound coenzyme seems unlikely. The X‐ray crystal structure of PETNR bound to NADH_4_ indicates the side chain of Arg130 faces the outer side of the active site channel and points away from the tail of the nicotinamide coenzyme. Specifically, the guanidino moiety of Arg130 is > 7 Å away from the pyrophosphate and > 10 Å away from the 2′‐hydroxyl group of bound NADH_4_. To investigate the interaction of Arg130 with NAD(P)H, the neutral variants R130M and R130L, along with the negatively charged variant R130E, were created. The RHR of each variant was characterized by stopped‐flow spectroscopy. The dependence of the observed rate of FMN reduction (*k*_obs_) on NADH and NADPH concentration at 25 °C was determined (Figs S1–S4), and the kinetic parameters obtained by fitting observed rate constants to Eq.  are shown in Fig. 2, along with previously reported values for wild‐type (WT) PETNR 49 represented for comparison.

(1) $$k_{\mathrm{obs}}=k_{\mathrm{rev}}+\frac{k_{\mathrm{red}}\;\left[\mathrm{NAD}\left(P\right)H\right]}{K_{S}+\left[\mathrm{NAD}\left(P\right)H\right]}$$

Wild‐type PETNR is reduced by both NADH and NADPH, with NADPH having a limiting rate constant (*k*_red_) value 17‐fold higher than NADH and 10‐fold higher affinity toward NADPH (*K*_S_ of 0.1 mm) than for NADH 49. Consequently, NADPH is the preferred coenzyme for WT PETNR, with an overall efficiency for performing the RHR (*k*_red_/*K*_S_) 170‐fold higher than NADH. However, by targeting Arg130 of PETNR, we observed significant coenzyme‐dependent changes to these kinetic parameters (Fig. 2). The values of all kinetic parameters for the reactions of R130L, R130M, and R130E PETNR with NADH are broadly maintained and the only notable differences are the presence of two kinetic phases for the reduction of FMN in R130L and R130M PETNR (Figs S1–S4; multiple kinetic phases attributed to conformational heterogeneity in the active site have been previously observed in other OYE ene‐reductases 49, 56) and a threefold decrease in NADH affinity (*K*_S_) and efficiency (*k*_red_/*K*_S_) for R130E. These results suggest that Arg130 does not have a major role in NADH binding to PETNR. It is likely that the modest changes observed in the kinetic parameters are a propagated effect of the substitution, that is, causing other nearby residues with a functional role to adopt a different conformation, or creating a slight electrostatic repulsion in the case of the R130E variant.

In contrast, both R130L and R130M PETNR variants show noticeably decreased rates of hydride transfer in their reaction with NADPH. A reverse rate of reaction (*k*_rev_ = 0.46 ± 0.11 s^−1^ for both variants) and a striking > 80‐fold reduction in affinity values toward the phosphorylated coenzyme are also observed. This reduces the efficiency of the RHR (*k*_red_/*K*_S_) to values similar to the reaction of WT, R130L, and R130M PETNR with NADH. As expected, the most pronounced effect on the binding affinity of NADPH to PETNR is seen for the R130E variant. In this case, the substitution of the positively charged guanidino group with a negatively charged carboxylate prevents formation of a stable complex. Instead, FMN reduction by NADPH in R130E PETNR is second order, with a rate constant of 0.09 ± 0.01 s^−1^·mm ^−1^ and approximate *k*_red_ value of 13.5 s^−1^ (assuming a maximum solubility of NADPH of 150 mm). The approximate upper limit of *k*_red_, used in this study, enables better comparison of the catalytic efficiency between variants exhibiting second‐order as opposed to saturation kinetics. This kind of decrease in binding affinity upon replacement of an active site arginine with a glutamate was previously observed in other NADPH‐dependent dehydrogenases 45.

Since NADH and NADPH are isostructural (except at the 2′‐hydroxyl/phosphate groups), the striking differences in affinity of the variants for these coenzymes indicates that Arg130 likely coordinates the 2′‐phosphate group of NADPH. Replacement of Arg130 with a neutral amino acid of similar length (Met or Leu) leads to no discrimination between the two coenzymes, as evidenced by the similar *k*_red_/*K*_S_ values for NADH and NADPH. This is mostly caused by changes in affinity, with rates of FMN reduction by NADPH being higher than those with NADH in all variants. This suggests the Arg130 site is mainly involved in preferentially binding the tail of NADPH, with limited effects on the positioning of the nicotinamide site for H‐transfer.

### Residue Arg142 in the β‐hairpin flap of PETNR coordinates the pyrophosphate group of the nicotinamide coenzymes

**Figure 3:**
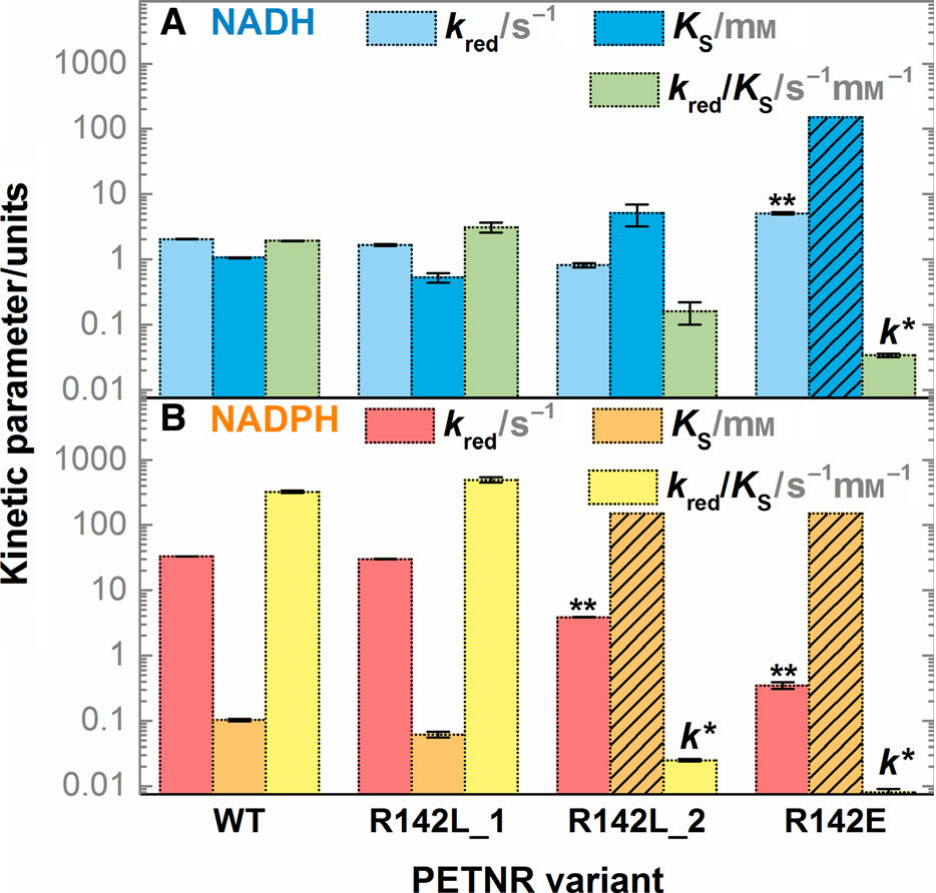
Kinetic parameters for the RHR of WT, R142L and R142E PETNR variants with (A) NADH and (B) NADPH. R142L_1 and R142L_2 denote the two different kinetic phases observed for the FMN reduction in R142L PETNR variant. The kinetic parameters are represented as bars, with the same *y*‐axis maintained in both panels for a better comparison. *In these cases, the kinetics of FMN reduction follow a second‐order reaction, with a rate constant, *k*, represented instead of the *k*_red_/*K*_S_ value. **k_red_ constants are approximate upper limit values, calculated by multiplying the second‐order rate constant (*k*) by the maximum solubility limit of NAD(P)H in solution (*K*_S_ ~ 150 mm), and the *K*_S_ value representing the solubility limit is shown as bars crossed out with black lines. Error bars are standard errors from the fit. All kinetic parameters are tabulated in Tables S1 and S2.

The R130E PETNR variant showing a moderate reduction in affinity toward NADH suggests that the R130E carboxylate may also perturb NADH and NADPH binding through electrostatic repulsion of the coenzyme pyrophosphate group. Significant NMR chemical shift perturbations of Ile141 (also in the β‐hairpin flap) have been observed on binding NADH_4_ 49, suggesting charged residue(s) in this loop are likely involved in coenzyme binding. The most likely candidate, Arg142, was targeted for mutagenesis, by substitution with Leu and Glu residues. The RHR of R142L and R142E variants was investigated using stopped‐flow spectroscopy (Figs S5–S6). Kinetic parameters from these measurements are presented in Fig. 3.

A notable feature of the reaction of R142L PETNR with both NADH and NADPH is the presence of two kinetic phases that contribute to the total change in amplitude at 465 nm. With NADH, one of the phases has similar kinetic parameters to WT PETNR. The other phase, which has a larger amplitude, has reduced *k*_red_ (0.82 ± 0.06 s^−1^) and increased *K*_S_ (5.07 ± 1.87 mm) values. This leads to significantly impaired reaction efficiency. The reaction of R142L with NADPH is similar, with a minor phase showing kinetic parameters that are comparable to WT PETNR and a dominant kinetic phase, which is apparently second order (*k *=* *0.025 ± 0.001 s^−1^·mm ^−1^). The RHR of R142E PETNR with NADH is monophasic and almost completely impaired, with FMN reduction following a second‐order reaction (*k *=* *0.034 ± 0.002 s^−1^), comparable to the dominant kinetic phase of R142L PETNR with NADPH. The reaction of R142E PETNR with NADPH is even more impaired (with a second‐order rate constant of 0.008 ± 0.002 s^−1^·mm ^−1^), consistent with an electrostatic clash between R142E and the NADPH 2′‐phosphate. For reference to the WT reactions, approximate maximal first‐order *k*_red_ values of 5 s^−1^ and 0.4 s^−1^ can be estimated for the RHRs of R142L PETNR with NADH and NADPH, respectively, at saturating (150 mm) concentrations of NAD(P)H.

Removal of the positive charge of the Arg142 side chain leads to notable reduction in the ability of the enzyme to bind both NADH and NADPH. These data are consistent with Arg142 stabilizing bound NAD(P)H through electrostatic interactions with the coenzyme pyrophosphate group. Differences between reactions of R142E PETNR with NADH and NADPH, and the multiple kinetic phases observed in the reactions of R142L PETNR, may arise through alternative (and poorly reactive) coenzyme‐binding conformations where the pyrophosphate group forms ionic bond(s) with R130. Again, these data are consistent with an induced fit mechanism.

### Learning from PETNR enables switching of coenzyme specificity in NADH‐dependent MR

**Figure 4:**
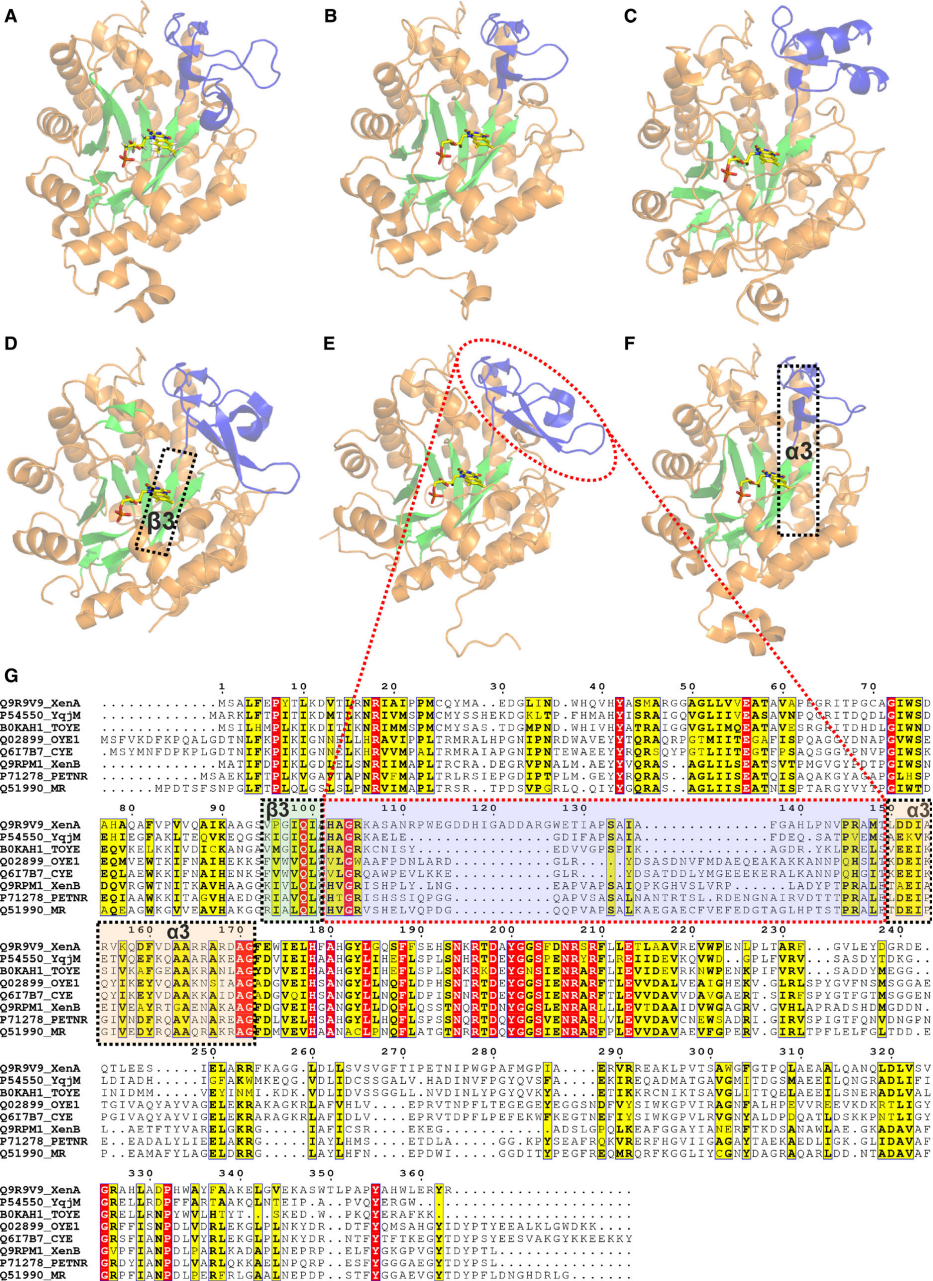
Structural architecture and multiple structural alignment of selected ene‐reductases from class I, II and III (as classified in 10) of the OYE family. The structures of several representative enzymes are shown: (A) XenA (PDB: 3L5L), (B) YqjM (PDB: 1Z41), (C) OYE1 (PDB: 1OYA), (D) PETNR (PDB: 5LGX), (E) MR (PDB: 1GWJ) and (F) TOYE (PDB: 3KRU). The structure of the OYEs is illustrated in cartoon form (colored orange, with the β‐sheets shown in green, highlighting the conserved TIM barrel architectural fold). The FMN cofactor is shown as yellow sticks, and the polypeptide excursion between the β3 strand and α3 helix of the TIM barrel is shown in blue. (G) Multiple sequence alignment of selected OYEs represented in (A)–(F), along with two more members (XenB and CYE) for which there are no available crystal structures. Residues highlighted in red are conserved among all selected OYEs, while those highlighted in yellow only partially conserved (sharing similar physico‐chemical properties). Each line on the first column of the figure is showing the Uniprot accession code followed by the abbreviated name of each ene‐reductase.

As previously mentioned, Arg130 is located in the β‐hairpin flap, which is part of a large polypeptide excursion situated between the β3 strand and α3 helix of the TIM barrel structure of PETNR (Figs 1 and 4). While this motif of the enzyme is flanked by two conserved regions across OYEs (the β3 strand and the α3 helix are essential secondary structure elements of the TIM barrel fold, see conservation of the structural features across the family in Fig. 4A–F), the sequence identity of this loop is not conserved between members of the OYE family (Fig. 4G), which explains why a common coenzyme‐binding sequence motif has not been identified for OYEs. This leads one to question if there are equivalent residue(s) to Arg130 in other OYEs, and if so, how would one identify these residue(s) to engineer new coenzyme selectivity?

**Figure 5:**
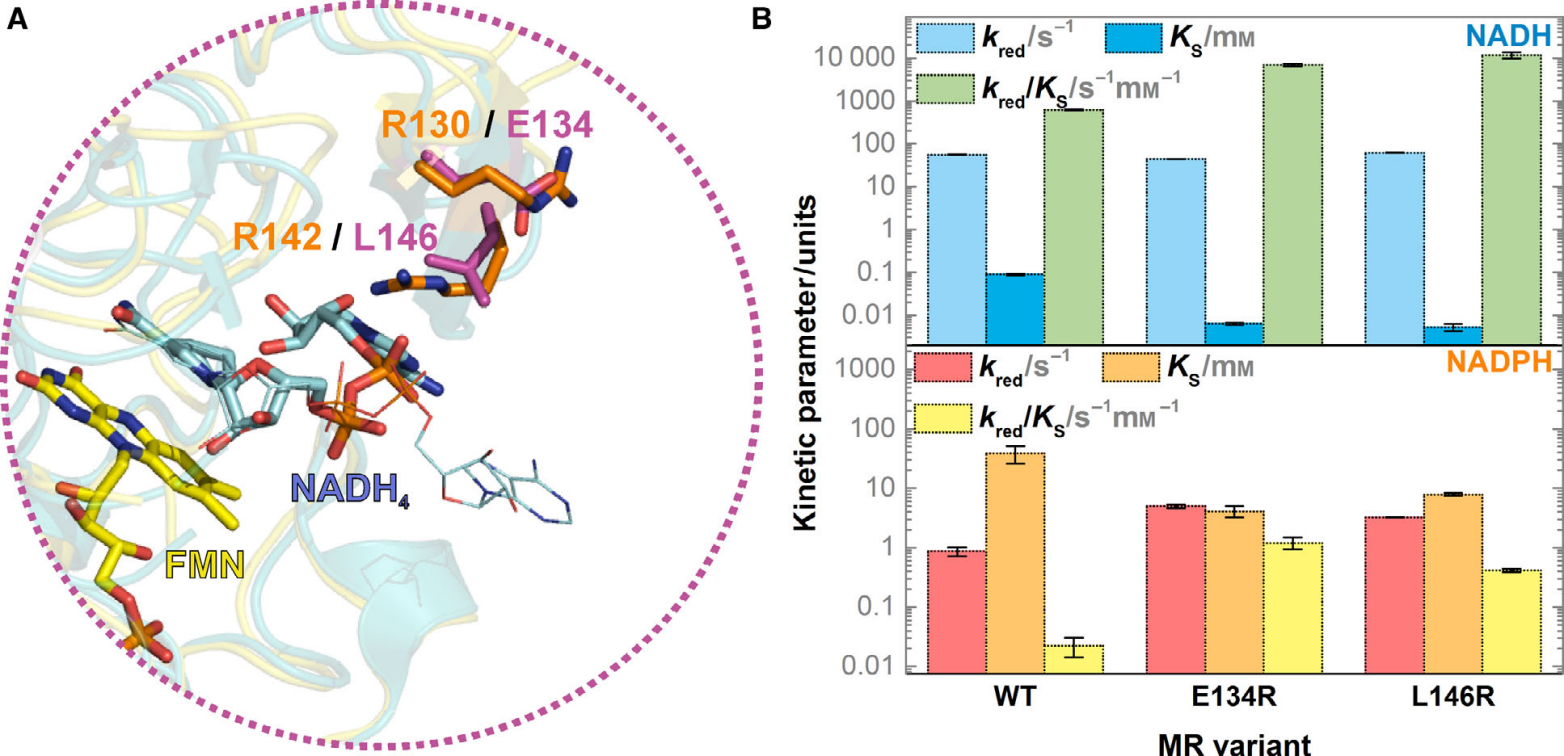
(A) Overlay of the X‐ray crystal structures of PETNR:NADH_4_ (teal cartoon, orange sticks, PDB: 3KFT) and MR:NADH_4_ complexes (yellow cartoon, magenta sticks, PDB: 2R14) showing the active site and β‐hairpin flap. The NADH_4_ conformation in PDB: 3KFT is displayed in line form. (B) Kinetic parameters for the RHR of WT, E134R, and L146R MR variants with NADH (top) and NADPH (bottom). The kinetic parameters are represented as bars, and error bars are standard errors from the fit. All kinetic parameters are tabulated in Tables S3 and S4.

MR is a dimer and it uses NADH in its natural catalytic cycle 42, 55. It shares 51% sequence identity with PETNR and the subunit X‐ray crystal structures of the two enzymes are similar. MR also has a β‐hairpin flap in a position similar to that found in PETNR. Comparison of the β‐hairpin region in PETNR and MR indicates that MR does not possess residues equivalent to Arg130 and Arg142 found in PETNR. Instead, MR possesses an acidic residue (Glu134) in place of Arg130, and the neutral side chain of Leu146 in place of Arg142 found in PETNR (Fig. 5A). As Arg130 governs coenzyme specificity in PETNR and R130E PETNR variant does not accept NADPH as a hydride donor, we reasoned that the NADH‐only specificity of MR might be attributed to the presence of Glu134. To test this hypothesis, we created two MR variants, E134R and L146R, and characterized their properties using stopped‐flow spectroscopy. The resulting kinetic parameters are presented in Fig. 5B.

The RHR of WT MR with NADH proceeds with *k*_red_ = 55.44 ± 0.41 s^−1^ and *K*_S_ = 89 ± 4 μm 53, 57. The limiting rate of reaction of E134R and L146R MR with NADH are similar to WT with *k*_red_ = 44.64 ± 0.07 and 61.69 ± 0.22 s^−1^, respectively. However, the introduction of an arginine at either site leads to a dramatic increase in binding affinity toward NADH, with estimated *K*_S_ values of 6 and 5 ± 1 μm for E134R and L146R, respectively. These are likely to be an upper limit due to the experimental limitations of the study (Fig. S7). Again, these results are consistent with coenzyme binding through ionic interactions between the NADH pyrophosphate moiety and the guanidine side chain(s) of arginine residues in the β‐hairpin flap. The small differences in H‐transfer rates (*k*_red_) between MR variants may be attributed to subtle perturbations of the nicotinamide moiety of the NADH in each Michaelis complex.

Finally, we investigated whether NADPH could reduce any of the MR variants. While it was previously reported that WT MR does not react with NADPH 42, we were able to observe slow RHR kinetics at very high concentrations of NADPH (*k*_red_ = 0.86 ± 0.15 s^−1^, *K*_S_ = 38.5 ± 12.6 mm). Along with this slow phase, a minor fast phase was observed, which we attribute to a small amount of NADH impurity (0.05–0.1%) in the NADPH stock (Fig. S8). The reactions of E134R and L146R MR with NADPH show greatly improved RHR kinetics, with reaction efficiencies (*k*_red_/*K*_S_) 55‐ and 20‐fold greater than for WT MR, respectively (Fig. 5B). As improvements in both *k*_red_ and *K*_S_ were observed, improved NADPH binding did not impair the rate of this reaction. Furthermore, the E134R and L146R MR variants also give rise to a 10‐ and 20‐fold increase in *k*_red_/*K*_S_ for the RHR with NADH, respectively, when compared to WT MR. Together, these results show that the introduction of an arginine residue at either of the targeted sites (Arg130 and Arg142 in PETNR) leads to improved RHR kinetics in MR.

This leads to the intriguing question as to why PETNR and MR have not evolved to bind nicotinamide coenzymes more tightly. First, there is likely to be no strong evolutionary constraint to achieve values *K*_S_ < 100 μm, as NADH is often detected at concentrations above 100 μm *in vivo* 58, 59. Second, tight binding of NAD(P)H may lead to tight binding of the NAD(P)^+^ product, preventing fast release from the active site. This would slow the overall rate of catalytic turnover, since the complete enzyme reaction cycle is a shared single‐site ping‐pong mechanism. As such, the β‐hairpin flap might also have a role in binding the oxidative substrate, and further improvement of NAD(P)H binding might be at the expense of binding productively the oxidative substrate in the enzyme active site.

To investigate this aspect, we investigated the steady‐state turnover kinetics of E134R and L146R MR with NADH and a widely used OYE family substrate, 2‐cyclohexen‐1‐one. Similar kinetics to WT MR 53, 55 were observed (Fig. S9) and the oxidative half‐reaction was rate‐limiting in all cases. This finding demonstrates that improved coenzyme affinity does not necessarily lead to impaired MR oxidative half‐reaction kinetics. As the E134R and L146R MR variants have enhanced NADH affinity and as well as an ability to work with NADPH, they offer new opportunities for use in biocatalysis applications.

We were able to dramatically change the affinity of the NADH‐dependent MR toward both NADH and NADPH by redesigning the β‐hairpin flap through single‐point mutagenesis. Based on these results, we suggest that coenzyme specificity can be rationally designed in other members of the OYE family by targeting the large polypeptide excursion between the β3 strand and the α3 helix of the TIM barrel. Although the sequence (and often the structural elements) of this loop is not conserved across the family, the large degree of sequence conservation/similarity of the flanking regions (in particular, of the β3 strand and the α3 helix and also of the first and last four residues of the loop element; Fig. 4) should enable a focused approach toward switching coenzyme specificity in other OYEs, which could be applied in the absence of crystallographic data as well.

## Concluding remarks

The construction of efficient metabolic pathways requires the ability to control enzymatic nicotinamide coenzyme utilization, but also to engineer or reverse coenzyme preference in oxidoreductases, one of the largest classes of enzymes frequently used in biocatalytic processes. Efforts have been made toward understanding and switching coenzyme preference of oxidoreductases, in particular for the dehydrogenase family 45, 47, 60, 61. Despite being targets for a large number of biocatalytic processes 9, 12, there is a lack of understanding regarding the molecular basis of NAD(P)H specificity in OYEs, the largest group of enzymes in the ene‐reductase class of oxidoreductases.

We have demonstrated that charged residues in the β‐hairpin flap of two OYEs are largely responsible for the tight and selective binding of nicotinamide coenzymes in these ene‐reductases. We have established that the β‐hairpin structural motif dictates the affinity of PETNR toward NADPH through electrostatic interactions between two arginine residues (Arg130 and Arg142) and both the pyrophosphate and 2′‐phosphate groups of the coenzyme. Inspection of conserved *structural* (not sequence) motifs in MR and PETNR identified two residues in MR (Glu134 and Leu146) that control binding affinity and coenzyme selectivity in this enzyme. Structure‐based design has therefore addressed long‐standing uncertainties related to coenzyme specificity in OYEs. The majority of OYEs have a similar loop emerging between strand β3 and helix α3 of the TIM barrel. While sequence similarity might not be conserved in this region, structure‐based approaches, as described herein, should allow tuning of nicotinamide affinity and selectivity in other members of the OYE family.

## Experimental section

### Materials

All commercial reagents were of analytical grade and were purchased from Sigma‐Aldrich (Dorset, UK), unless otherwise stated. NADH and NADPH were procured from Melford Laboratories (Chelsworth, UK).

### Cloning, overexpression, and purification of variant enzymes

Pentaerythritol tetranitrate reductase from *Enterobacter Cloacae PB2* and MR from *Pseudomonas putida* were overexpressed from C‐terminal His_6_‐tagged constructs cloned into pET21a plasmids. The desired mutations were introduced into PETNR and MR genes using the Q5 Site‐Directed Mutagenesis Kit from New England BioLabs (Hitchin, UK), with custom primers ordered from Eurofins Genomics (Ebersberg, Germany). The designed nonoverlapping primers used for each variant are presented in Table S5. All mutations were confirmed by DNA sequencing (Eurofins Genomics). NiCo21(DE3) *Escherichia coli* cells were used for overexpression of all variant enzymes. The His_6_‐tagged enzymes were isolated by affinity chromatography, using HisTrap HP nickel‐charged IMAC columns from GE Healthcare (Little Chalfont, UK).

### Extinction coefficients

NADH and NADPH concentrations were determined using a molar extinction coefficient of 6.22 mm ^−1^·cm^−1^ at 340 nm 62. PETNR and MR enzymes concentrations were determined using a molar extinction coefficient of 13.3 mm ^−1^·cm^−1^ at 46 nm (the same value was used for all enzyme variants, as they presented the same UV‐vis spectral features as the WT enzyme, with mutagenesis not affecting the characteristic spectra of PETNR/MR‐bound FMN).

### Stopped‐flow spectroscopy

The RHR of the ene‐reductases (PETNR and MR) with NADH and NADPH was investigated using a Hi‐Tech Scientific (TgK Scientific, Bradford on Avon, UK) stopped‐flow spectrophotometer, which had the sample handling unit placed inside a Belle Technology anaerobic glovebox (<5 p.p.m. of O_2_). All experiments were performed in 50 mm potassium phosphate buffer solution, pH 7.0, which was degassed prior to the experiments, as previously described 49. All concentration dependence measurements were performed at 25 °C, using 20 μm enzyme (final concentration, after mixing of the two reactant solutions) and various NADPH or NADH concentrations (7–12 different concentrations for each concentration dependence experiment, 0.1–25 mm final coenzyme concentration). FMN reduction was observed by continuously monitoring the decrease in absorbance at 465 nm (maximum peak for oxidized flavin‐bound enzyme, same for both PETNR and MR). All transient kinetic traces were analyzed and fitted with standard first‐, second‐, or third‐order exponential decay functions (depending on the number of phases observed), using originpro 9.1 (OriginLab Corporation, MA, USA). The reported observed rate constants (*k*_obs_) represent the mean average of three to six individual measurements, with error bars plotted as ± 1 standard deviation. The limiting rate constant (*k*_red_) and the apparent saturation constant (*K*_S_) for the RHR of each variant with NAD(P)H were determined by fitting the *k*_obs_ values at varying coenzyme concentration to a hyperbolic function (Eq. 1).

### Steady‐state kinetics

The reduction of 2‐cyclohexen‐1‐one using MR variants was followed by monitoring the oxidation of NADH (marked by the decrease in absorbance at 340 nm). All measurements were carried out at 25 °C in 50 mm potassium phosphate buffer solution, pH 7.0, using a saturating concentration of NADH (200 μm). All experiments were performed anaerobically, using a Hi‐Tech Scientific stopped‐flow spectrophotometer, by mixing a reactant solution consisting of 0.2 μm enzyme and 150 μm NADH (prepared prior to the stopped‐flow mixing) with a reactant solution containing variable concentrations (0.5–50 mm) of 2‐cyclohexen‐1‐one. All 2‐cyclohexen‐1‐one reactant solutions were freshly prepared just before use, and three to six traces were recorded for each substrate concentration. All transient kinetic traces were fitted with a standard linear function, using OriginPro 9.1. The maximum velocity (*V*_max_) and the Michaelis constant (*K*_M_) were determined by fitting the initial reaction rates at varying 2‐cyclohexen‐1‐one concentrations to the Michaelis–Menten equation (Eq. 2).

(2) $$V_{0}=\frac{V_{\max}\;\left[2\text{-}\mathrm{cyclohexen}\text{-}1\text{-}\mathrm{one}\right]}{K_{M}+\left[2\text{-}\mathrm{cyclohexen}\text{-}1\text{-}\mathrm{one}\right]}$$

### Multiple sequence alignment

The multiple sequence alignment was performed using the Clustal Omega web server 63 and the alignment file was rendered using the ENDscript server 64.

## Conflict of interest

The authors declare no conflict of interest.

## Author contributions

AII designed and performed the experiments. AII and TMH analyzed data. All authors discussed the results. AII wrote the paper, with input from all authors. SH and NSS supervised the work.

## Supporting information


**Fig. S1.** Concentration dependence of FMN reduction in WT PETNR with NADH and NADPH.
**Fig. S2.** Concentration dependence of FMN reduction in R130L PETNR variant with NADH and NADPH.
**Fig. S3.** Concentration dependence of FMN reduction in R130M PETNR variant with NADH and NADPH.
**Fig. S4.** Concentration dependence of FMN reduction in R130E PETNR variant with NADH and NADPH.
**Fig. S5.** Concentration dependence of FMN reduction in R142L PETNR variant with NADH and NADPH.
**Fig. S6.** Concentration dependence of FMN reduction in R142E PETNR variant with NADH and NADPH.
**Fig. S7.** Concentration dependence of FMN reduction in WT MR, E134R MR, and L146R MR variants with NADH.
**Fig. S8.** Concentration dependence of FMN reduction in WT MR, E134R MR, and L146R MR variants with NADPH.
**Fig. S9.** Steady‐state kinetics for the reduction in 2‐cyclohenexen‐1‐one with WT, E134R and L146R MR variants.
**Table S1.** Kinetic parameters for the reductive half‐ reaction of PETNR variants with NADH.
**Table S2.** Kinetic parameters for the reductive half‐ reaction of PETNR variants with NADPH.
**Table S3.** Kinetic parameters for the reductive half‐ reaction of MR variants with NADH.
**Table S4.** Kinetic parameters for the reductive half‐reaction of MR variants with NADPH.
**Table S5.** Forward and reverse primers sequences used for site‐directed mutagenesis of PETNR and MR.
