# Supplementary material for: Selectivity through discriminatory induced fit enables switching of NAD(P)H coenzyme specificity in Old Yellow Enzyme ene‐reductases
Source: FEBS J. 2019 May 13;286(16):3117–28. doi: 10.1111/febs.14862 (PMC6767020; doi:10.1111/febs.14862)
Supplement: Supplementary file 1 — Fig. S1. Concentration dependence of FMN reduction in WT PETNR with NADH and NADPH. Fig. S2. Concentration dependence of FMN reduction in R130L PETNR variant with NADH and NADPH. Fig. S3. Concentration dependence of FMN reduction in R130M PETNR variant with NADH and NADPH. Fig. S4. Concentration dependence of FMN reduction in R130E PETNR variant with NADH and NADPH. Fig. S5. Concentration dependence of FMN reduction in R142L PETNR variant with NADH and NADPH. Fig. S6. Concentration dependence of FMN reduction in R142E PETNR variant with NADH and NADPH. Fig. S7. Concentration dependence of FMN reduction in WT MR, E134R MR, and L146R MR variants with NADH. Fig. S8. Concentration dependence of FMN reduction in WT MR, E134R MR, and L146R MR variants with NADPH. Fig. S9. Steady‐state kinetics for the reduction in 2‐cyclohenexen‐1‐one with WT, E134R and L146R MR variants. Table S1. Kinetic parameters for the reductive half‐ reaction of PETNR variants with NADH. Table S2. Kinetic parameters for the reductive half‐ reaction of PETNR variants with NADPH. Table S3. Kinetic parameters for the reductive half‐ reaction of MR variants with NADH. Table S4. Kinetic parameters for the reductive half‐reaction of MR variants with NADPH. Table S5. Forward and reverse primers sequences used for site‐directed mutagenesis of PETNR and MR. [file FEBS-286-3117-s001.zip › febs14862-sup-0001-SupInfo.pdf]

SUPPORTING INFORMATION:

SELECTIVITY THROUGH DISCRIMINATORY INDUCED FIT ENABLES SWITCHING OF NAD(P)H COENZYME SPECIFICITY IN OLD YELLOW ENZYME ENE-REDUCTASES

*Andreea I. Iorgu, Tobias M. Hedison, Sam Hay, Nigel S. Scrutton**

Manchester Institute of Biotechnology and School of Chemistry, Faculty of Science and Engineering,

The University of Manchester, 131 Princess Street, Manchester M1 7DN, United Kingdom

nigel.scrutton@manchester.ac.uk

TABLE OF CONTENTS

NADH AND NADPH CONCENTRATION DEPENDENCE STUDIES FOR THE RHR OF PETNR VARIANTS S2

NADH AND NADPH CONCENTRATION DEPENDENCE STUDIES FOR THE RHR OF MR VARIANTS S5

STEADY-STATE KINETICS FOR THE REACTION OF MR VARIANTS WITH 2-CYCLOHEXEN-1-ONE...S7

TABULATED KINETIC PARAMETERS FOR THE RHR OF PETNR AND MR VARIANTS S8

PRIMERS SEQUENCES USED FOR MUTAGENESIS S10

REFERENCES S11

NADH AND NADPH CONCENTRATION DEPENDENCE STUDIES FOR THE RHR OF PETNR VARIANTS

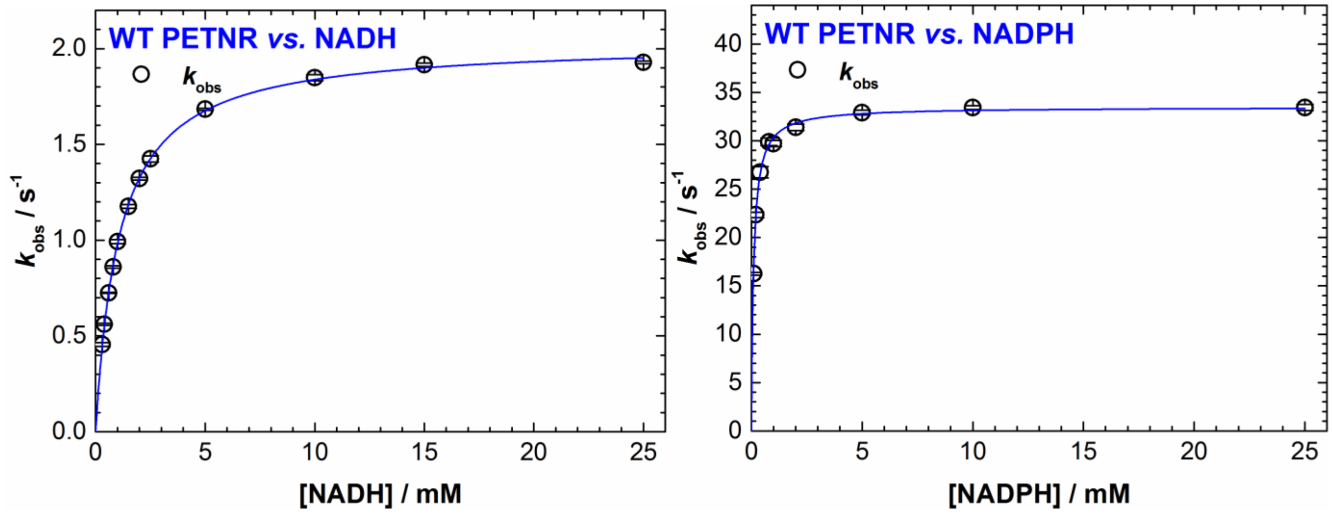

Fig. S1. Concentration dependence of FMN reduction in WT PETNR variant with NADH and NADPH – previously reported [1].

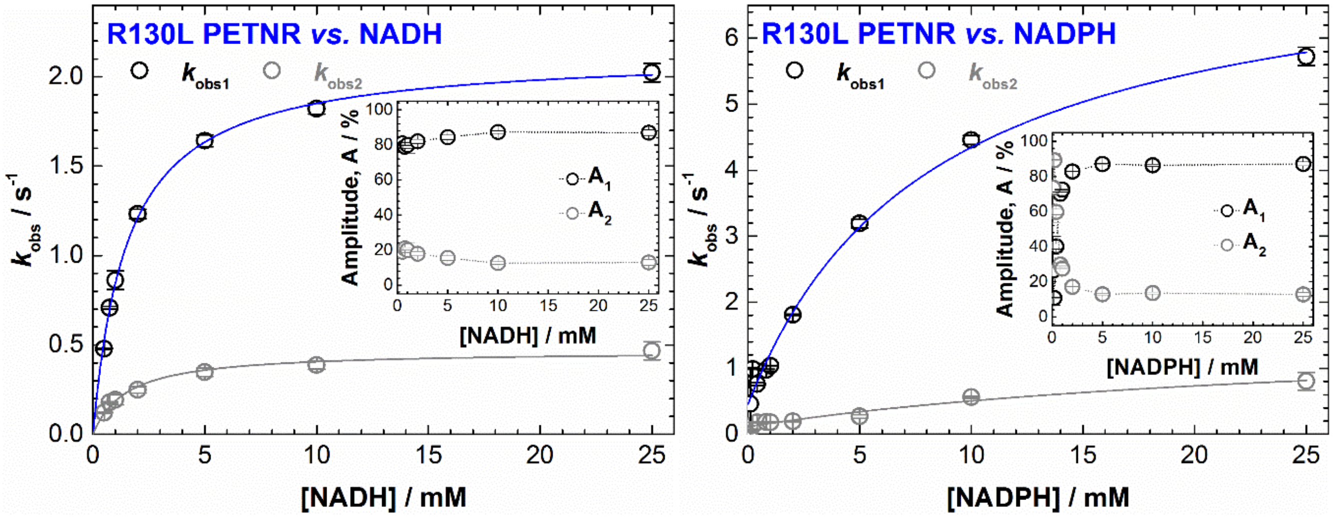

Fig. S2. Concentration dependence of FMN reduction in R130L PETNR variant with NADH and NADPH. *Inserts:* Concentration dependence of the amplitude of each observed kinetic phase. *Note:* the reactions are biphasic, and the observed rate constants for both phases could be fitted to a hyperbolic function. For the NADH reaction, the amplitudes of both phases remain largely unaffected throughout the whole concentration range, with values of 83 ± 3 % for A_1 and 17 ± 3 % for A_2, and similar K_S values of ~ 1.5 mM for both kinetic phases. For the NADPH reaction, the amplitude of the slow phase (k_{red2} = 1.47 ± 0.5 s-1, K_{S2} = 27.6 ± 16.4 mM) decreases with increasing NADPH concentration, with the fast phase being more dominant for the majority of the concentration range studied.

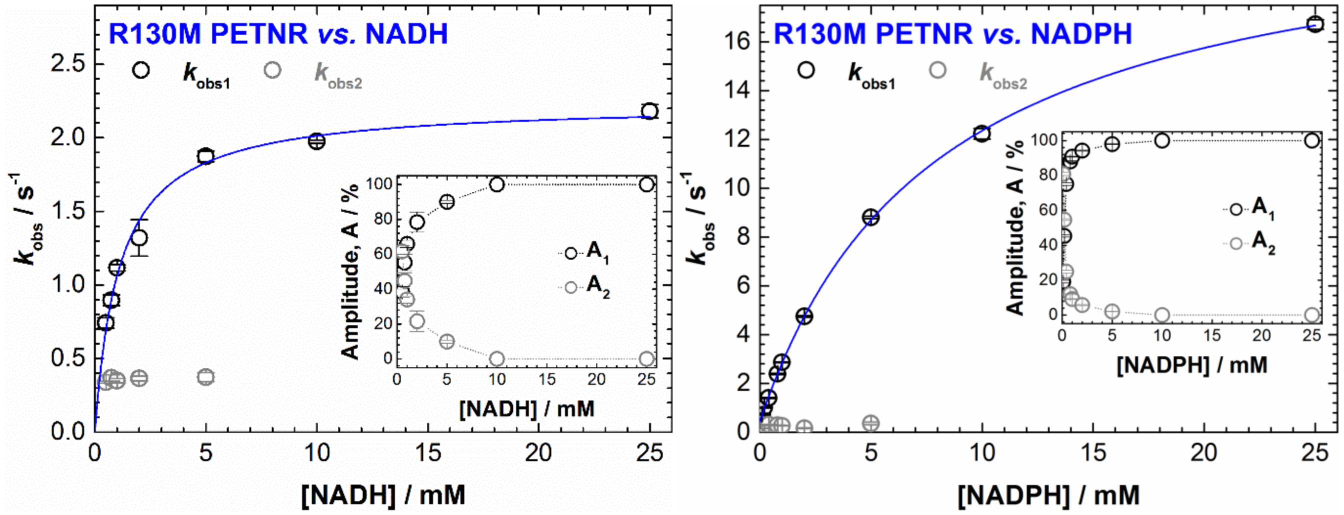

Fig. S3. Concentration dependence of FMN reduction in R130M PETNR variant with NADH and NADPH. *Inserts:* Concentration dependence of the amplitude of each observed kinetic phase. *Note:* the reactions are biphasic, with the slow phase (averaging 0.36 ± 0.01 s^{-1} and 0.26 ± 0.07 $s^{-}1$ for NADH and NADPH reactions, respectively) manifesting a decrease in amplitude with an increase in coenzyme concentration; for both reactions, only the fast phase can be observed at coenzyme concentrations higher than the K_S of the fast phase.

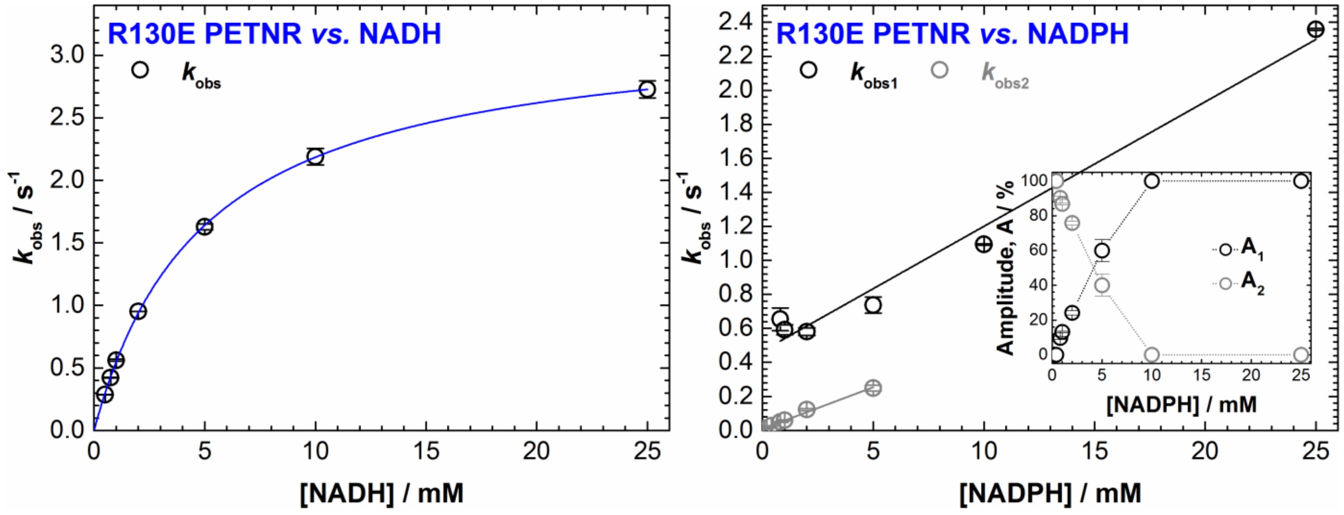

Fig. S4. Concentration dependence of FMN reduction in R130E PETNR variant with NADH and NADPH. *Insert for the left panel:* Concentration dependence of the amplitude of each observed kinetic phase. *Note:* for the NADPH reaction, both phases could only be fitted to a linear function, suggesting the reaction follows second-order kinetics. Second-order rate constants (k) were determined from the slope of the linear fit of the concentration dependence of the observed rate constants and a theoretical k_{red} constant was calculated by multiplying k by the maximum solubility limit of NADPH in solution (K_S ~ 150 mM).

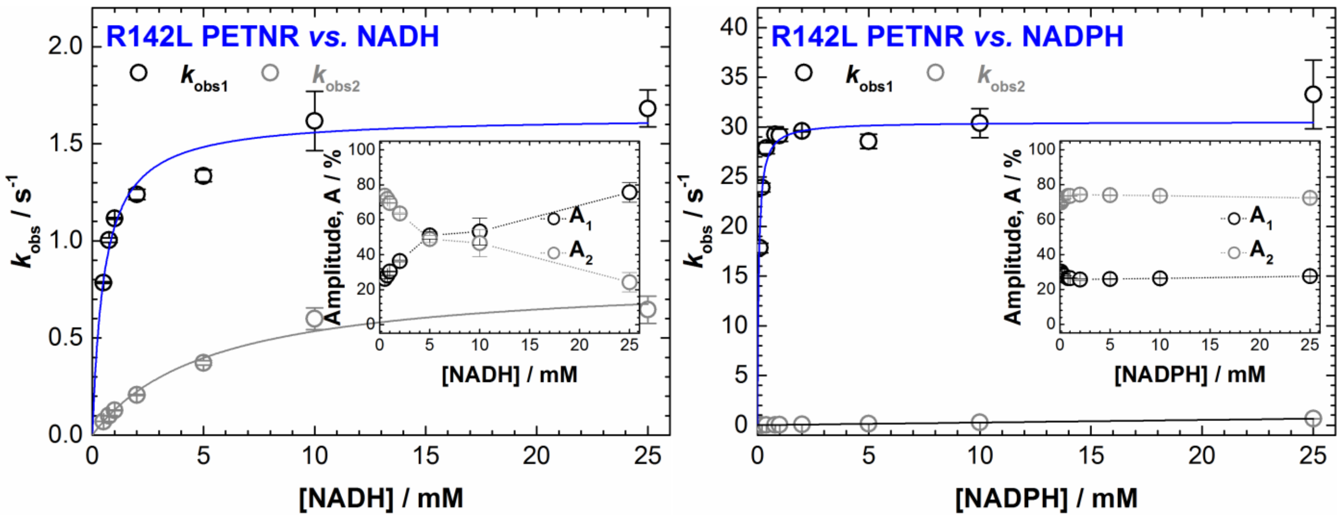

Fig. S5. Concentration dependence of FMN reduction in R142L PETNR variant with NADH and NADPH. *Inserts:* Concentration dependence of the amplitude of each observed kinetic phase. *Note:* the reactions are biphasic, and the observed rate constants for both phases could be fitted to hyperbolic/linear functions. For the NADH reaction, the amplitude of the slow phase (k_{red2} = 0.81 s^{-1}, K_{S2} = 5.3 ± 1.0 mM) decreases with increasing NADH concentration. For the NADPH reaction, the amplitudes of both phases remain largely unaffected throughout the whole concentration range, with values of 27 ± 2 % for A_1 and 73 ± 2 % for A_2 (the fast phase shows similar kinetic parameters to WT PETNR, while the slow dominant phase follows the kinetics of a second-order reaction, with k = 0.026 ± 0.001 s^{-1}).

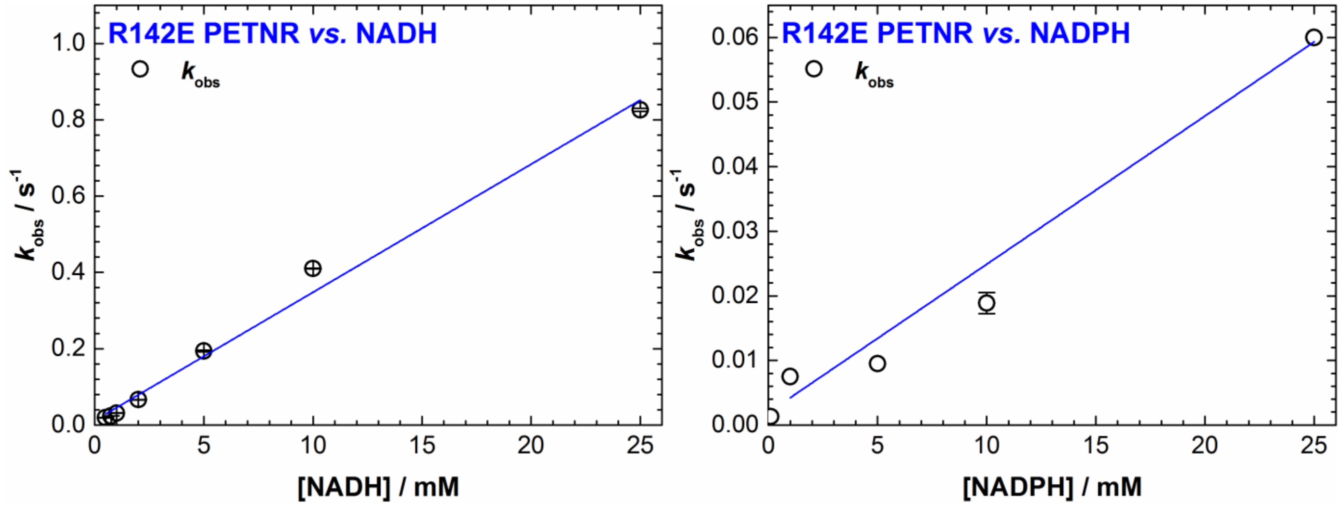

Fig. S6. Concentration dependence of FMN reduction in R142E PETNR variant with NADH and NADPH. *Note:* the observed rate constants could only be fitted to a linear function, suggesting the reaction follows second-order kinetics. Second-order rate constants (k) were determined from the slope of the linear fit of the concentration dependence of the observed rate constants and a theoretical k_{red} constant was calculated by multiplying k by the maximum solubility limit of coenzyme in solution (K_S ~ 150 mM).

NADH AND NADPH CONCENTRATION DEPENDENCE STUDIES FOR THE RHR OF MR VARIANTS

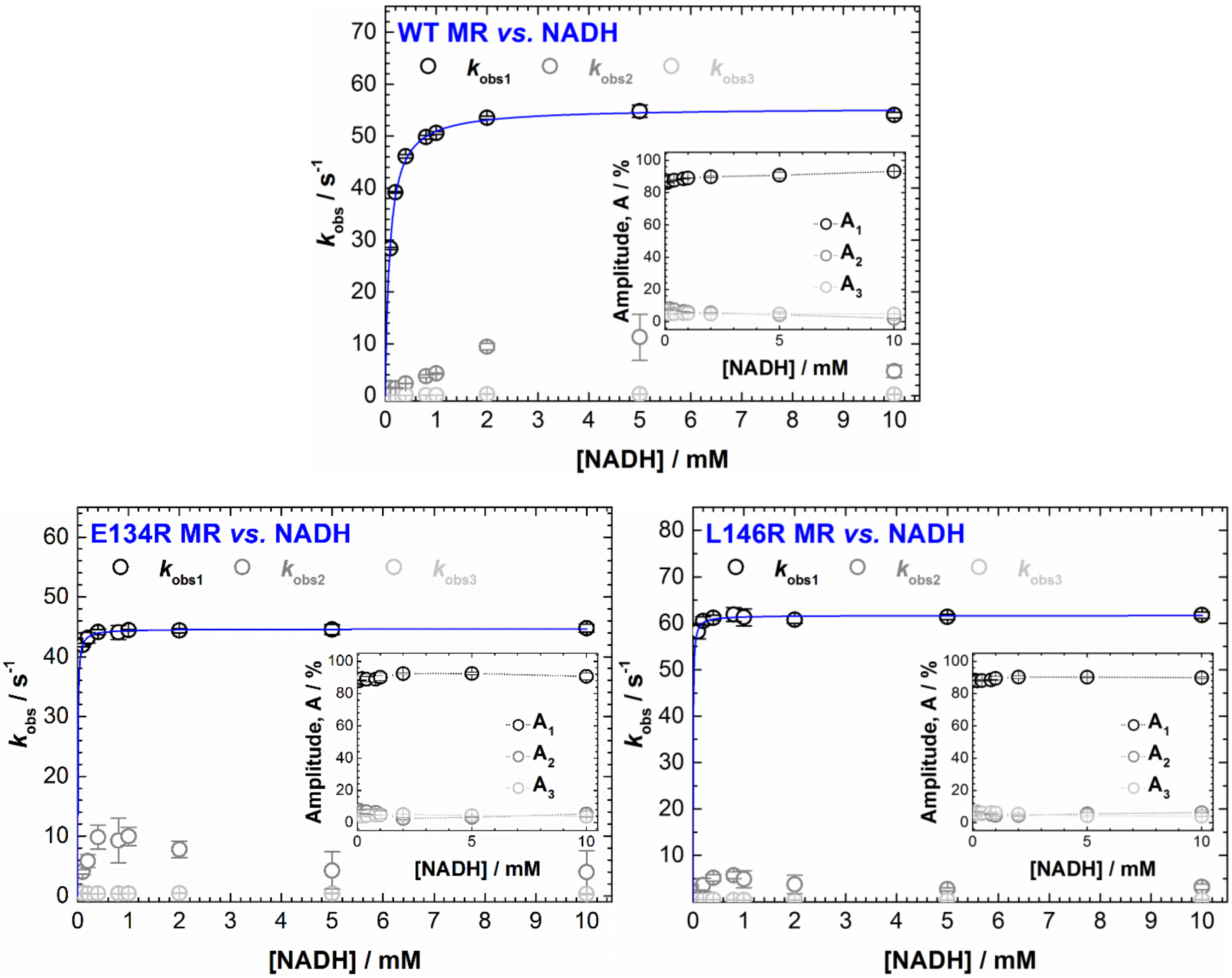

Fig. S7. Concentration dependence of FMN reduction in WT MR, E134R MR and L146R MR variants with NADH. The reactions present multiphasic behavior, as previously reported [2]. The two slow rates (k_{obs2} and k_{obs3}) contribute only to about 10% of the total amplitude change throughout the whole coenzyme concentration range (and are, most probably, non-catalytic). All kinetic parameters are tabulated in Table S3.

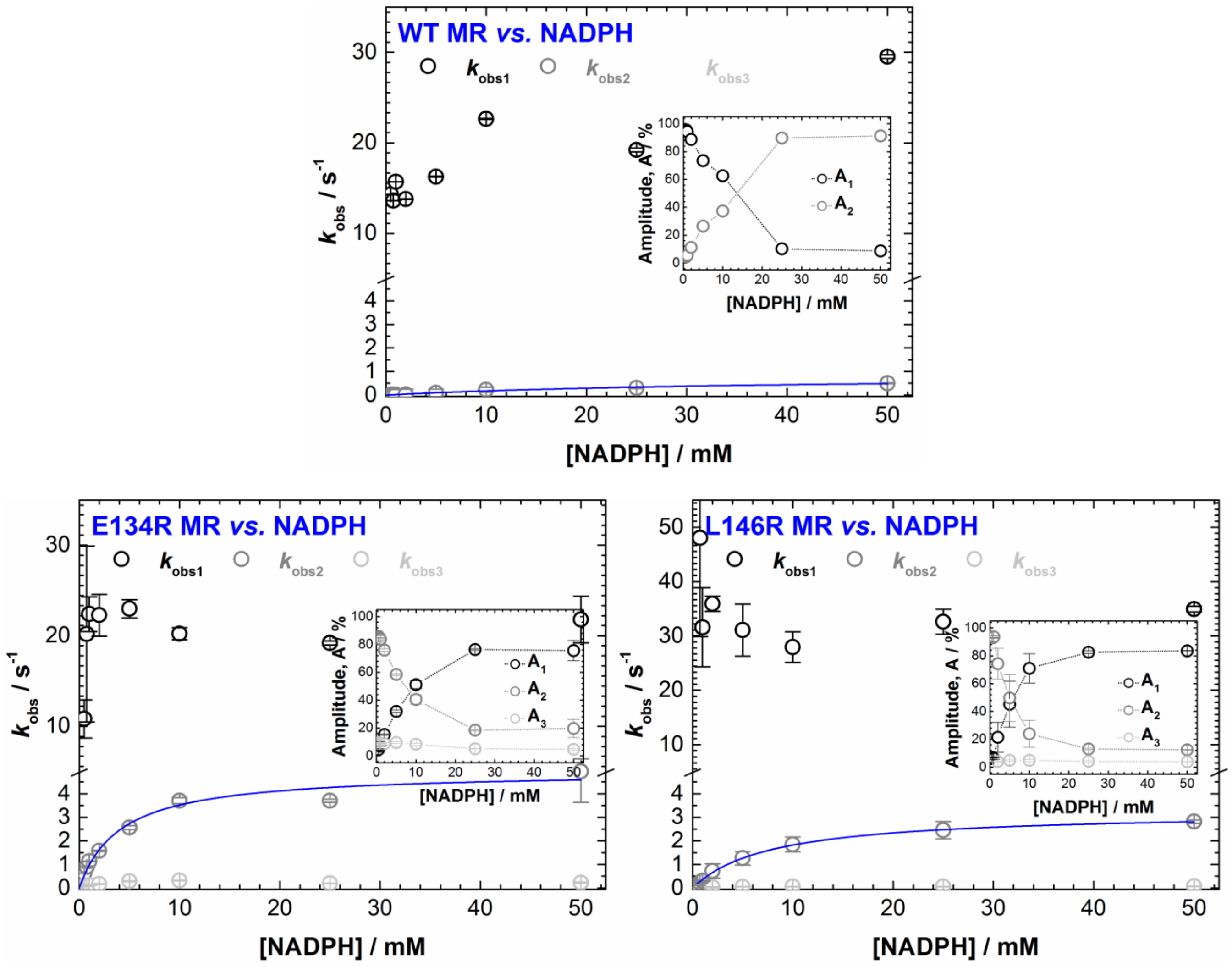

Fig. S8. Concentration dependence of FMN reduction in WT MR, E134R MR and L146R MR variants with NADPH. The reaction present multiphasic behavior, with up to three observed rate constants determined. The very slow rate (k_{obs3}) contributes only to about 10% of the total amplitude change throughout the whole coenzyme concentration range. The fast phase observed for WT MR (with an average k_{obs1} value of 18.13 ± 5.54 s^{-1} and no detectable K_S value) shows a linear increase in contribution to the total change in amplitude with increase in NADPH concentration. A similar phase was observed also in the reaction of NADPH with the other two variants (E134R and L146R), showing the same linear trend of increased contribution with increasing coenzyme concentration. Apart from a highly complex mechanism, which could not be readily explained, by applying Ockham's razor as a principle, a reasonable explanation for the presence of this phase is the contamination of the NADPH stock with a very small amount on NADH (~0.1%) impurity (which would explain the linear increase in contribution with increase in NADPH concentration and, thereby, of NADH impurity; moreover, the rates are similar to the k_{red} values describing the reaction of the variants with NADH, and the very tight binding of NADH to all the variants supports the observed results). All kinetic parameters are tabulated in Table S4.

STEADY-STATE KINETICS FOR THE REACTION OF MR VARIANTS WITH 2-CYCLOHEXEN-1-ONE

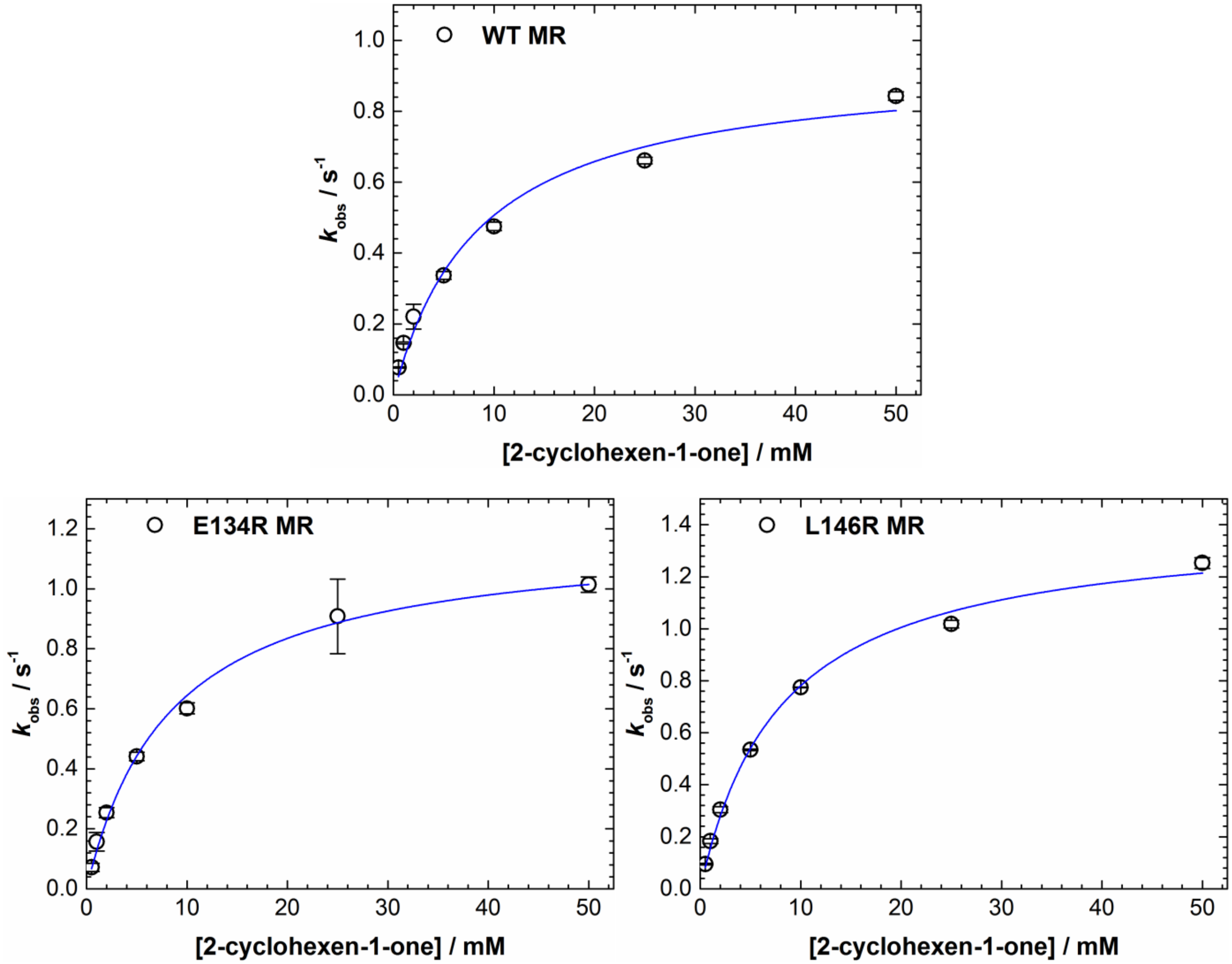

Fig. S9. Steady-state kinetics for the reduction of 2-cyclohenexen-1-one with WT, E134R and L146R MR variants. Fitting the observed rate constants to the Michaelis-Menten equation yields similar kinetic parameter values for all three variants (WT MR: k_{cat} = 0.93 ± 0.06 s-1, K_M = 8.54 ± 1.64 mM, E134R MR: k_{cat} = 1.18 ± 0.04 s-1, K_M = 8.37 ± 0.84 mM, L146R MR: k_{cat} = 1.41 ± 0.05 s-1, K_M = 8.04 ± 0.77 mM, with the values being comparable to previously published data [3,4]).

TABULATED KINETIC PARAMETERS FOR THE RHR OF PETNR AND MR VARIANTS

Table S1. Kinetic parameters for the reductive half-reaction of PETNR variants with NADH.

PETNR variant	k_{red} (s^{-1})	K_S (mM)	k_{rev} (s^{-1})	k_{red}/K_S (s^{-1} mM^{-1})	k (s^{-1} mM^{-1})	Additional comments
WT	2.03 ± 0.01	1.07 ± 0.01	-	1.90 ± 0.03	-	-
R130L	2.13 ± 0.03	1.53 ± 0.03	-	1.40 ± 0.06	-	2 phases*
R130M	2.24 ± 0.06	1.11 ± 0.10	-	2.01 ± 0.19	-	2 phases*
R130E	3.27 ± 0.02	4.95 ± 0.08	-	0.66 ± 0.01	-	-
R142L_1	1.64 ± 0.06	0.53 ± 0.09	-	3.09 ± 0.53		2 phases*
R142L_2	0.82 ± 0.06	5.07 ± 1.87	-	0.16 ± 0.06	-	-
R142E	-	-	-	-	0.034 ± 0.002	2nd-order reaction*

*For more details, see relevant supplementary figures above.

Table S2. Kinetic parameters for the reductive half-reaction of PETNR variants with NADPH.

PETNR variant	k_{red} (s^{-1})	K_S (mM)	k_{rev} (s^{-1})	k_{red}/K_S (s^{-1} mM^{-1})	k (s^{-1} mM^{-1})	Additional comments
WT	33.43 ± 0.22	0.103 ± 0.004	-	324.6 ± 12.8	-	-
R130L	7.08 ± 0.45	8.20 ± 1.54	0.46 ± 0.11	0.9 ± 0.2	-	2 phases*
R130M	21.39 ± 0.18	7.97 ± 0.21	0.46 ± 0.05	2.7 ± 0.07	-	2 phases*
R130E	-	-	-	-	0.09 ± 0.01	2nd-order reaction*
R142L_1	30.51 ± 0.15	0.061 ± 0.006	-	496.5 ± 47.2		2 phases*
R1422L_2	-	-	-	-	0.025 ± 0.001	
R142E	-	-	-	-	0.002 ± 0.001	2nd-order reaction*

*For more details, see relevant supplementary figures above.

Table S3. Kinetic parameters for the reductive half-reaction of MR variants with NADH.

**MR variant**	k_{red} (s^{-1})	K_S (mM)	k_{red}/K_S (s^{-1} mM^{-1})	k_{obs2}* (s^{-1})	k_{obs3}* (s^{-1})
WT	55.44 ± 0.41	0.089 ± 0.004	623.7 ± 29.9	4.85 ± 3.63	0.17 ± 0.09
E134R	44.64 ±0.07	0.006 ± 0.001	6899.7 ± 472.5	2.40 ± 1.60	0.19 ± 0.09
L146R	61.69 ± 0.22	0.005 ± 0.001	11682.9 ± 2084.2	3.93 ± 1.22	0.43 ± 0.05

*k_{obs2} and k_{obs3} are average values observed throughout the whole concentration range, contributing by ~ 5% each to the total change in amplitude at 465 nm.

Table S4. Kinetic parameters for the reductive half-reaction of MR variants with NADPH.

**MR variant**	k_{red}* (s^{-1})	K_S* (mM)	k_{red}/K_S (s^{-1} mM^{-1})	k_{obs1}** (s^{-1})	k_{obs3}*** (s^{-1})
WT	0.86 ± 0.15	38.5 ± 12.6	0.022 ± 0.008	18.13 ± 5.54	-
E134R	4.96 ±0.31	4.09 ± 0.88	1.21 ± 0.27	19.98 ± 3.93	0.18 ± 0.09
L146R	3.25 ± 0.06	7.85 ± 0.44	0.41 ± 0.02	38.17 ± 11.78	0.05 ± 0.01

*calculated from the hyperbolic fit of k_{obs2} values; **k_{obs1} is proposed to be a phase observed due to the presence of a small amount of NADH impurity in the NADPH stock (see more details in the Figure S14 caption); ***similar phase observed in the reaction with NADH.

PRIMERS SEQUENCES USED FOR MUTAGENESIS

Table S5. Forward and reverse primers sequences used for site-directed mutagenesis of PETNR and MR.

Enzyme variant	Forward primer sequence	Reverse primer sequence
R130L PETNR	5′- **CTG**ACTTCCCTGCGCGATGAAAAC -3′	5′- GGTATTGGCGTTCAGGGCAGAGGC-3′
R130M PETNR	5′- **ATG**ACTTCCCTGCGCGATGAAAAC-3′	5′- GGTATTGGCGTTCAGGGCAGAGGC-3′
R130E PETNR	5′- **GAA**ACTTCCCTGCGCGATGAAAAC-3′	5′- GGTATTGGCGTTCAGGGCAGAGGC-3′
R142L PETNR	5′- **CTG**GTCGACACCACCACGCCAC-3′	5′- GATCGCATTACCGTTTTCATCGCG-3′
R142E PETNR	5′- **GAA**GTCGACACCACCACGCCA-3′	5′- GATCGCATTACCGTTTTCATCGCG-3′
E134R MR	5′- **CGT**TGCTTTGTCGAATTCGAA-3′	5′- CGCACCCTCAGCTTTCAGTG-3′
L146R MR	5′- **CGT**CATCCGACCTCTACGCCGC-3′	5′- ACCTGCGGTGCCGTCTTCGAA-3′

REFERENCES

1 Iorgu AI, Baxter NJ, Cliff MJ, Levy CW, Waltho JP, Hay S & Scrutton NS (2018) Nonequivalence of Second Sphere "Noncatalytic" Residues in Pentaerythritol Tetranitrate Reductase in Relation to Local Dynamics Linked to H⁻Transfer in Reactions with NADH and NADPH Coenzymes. *ACS Catal.* **8**, 11589−11599.

2 Basran J, Harris RJ, Sutcliffe MJ & Scrutton NS (2003) H-tunneling in the multiple H-transfers of the catalytic cycle of morphinone reductase and in the reductive half-reaction of the homologous pentaerythritol tetranitrate reductase. *J. Biol. Chem.* **278**, 43973–43982.

3 Basran J, Harris RJ, Sutcliffe MJ & Scrutton NS (2003) H-tunneling in the Multiple H-transfers of the Catalytic Cycle of Morphinone Reductase and in the Reductive Half-reaction of the Homologous Pentaerythritol Tetranitrate Reductase. *J. Biol. Chem.* **278**, 43973–43982.

4 Barna T, Messiha HL, Petosa C, Bruce NC, Scrutton NS & Moody PCE (2002) Crystal Structure of Bacterial Morphinone Reductase and Properties of the C191A Mutant Enzyme. *J. Biol. Chem.* **277**, 30976–30983.
